# Promoting Well-Being: The Contribution of Emotional Intelligence

**DOI:** 10.3389/fpsyg.2016.01182

**Published:** 2016-08-17

**Authors:** Annamaria Di Fabio, Maureen E. Kenny

**Affiliations:** ^1^Department of Psychology, University of Florence, FlorenceItaly; ^2^Lynch School of Education, Boston College, Boston, MAUSA

**Keywords:** ability based emotional intelligence, trait emotional intelligence, hedonic well-being, eudaimonic well-being, primary prevention perspective, health promotion, healthy business, healthy organization

## Abstract

Adopting a primary prevention perspective, this study examines competencies with the potential to enhance well-being and performance among future workers. More specifically, the contributions of ability-based and trait models of emotional intelligence (EI), assessed through well-established measures, to indices of hedonic and eudaimonic well-being were examined for a sample of 157 Italian high school students. The Mayer–Salovey–Caruso Emotional Intelligence Test was used to assess ability-based EI, the Bar-On Emotional Intelligence Inventory and the Trait Emotional Intelligence Questionnaire were used to assess trait EI, the Positive and Negative Affect Scale and the Satisfaction With Life Scale were used to assess hedonic well-being, and the Meaningful Life Measure was used to assess eudaimonic well-being. The results highlight the contributions of trait EI in explaining both hedonic and eudaimonic well-being, after controlling for the effects of fluid intelligence and personality traits. Implications for further research and intervention regarding future workers are discussed.

## Introduction

As a construct of long-standing interest in the field of psychology, well-being deserves additional attention for its primary prevention potential for fostering health and performance in the workplace (Zelenski et al., 2008; Heuvel et al., 2010). While mental health professionals focus on restoring a state of well-being to workers who suffer from psychological distress, primary prevention and health promotion professionals seek to promote well-being and enhance factors that protect individuals from the negative effects of psychological risk (Hage et al., 2007). Interest in study of health promotion and well-being is expanding as numerous social, economic, biological, psychological and cultural factors pose threats to the attainment of well-being. In Italy, for example, young people as future workers are growing up in an era of rapid social change, job instability, and high unemployment. Economic insecurity, unstable and shifting work opportunities, and the subsequent increase in the number of people living in poverty pose risks to well-being for many youth and young adults (Masten, 2014; Di Fabio and Bucci, 2016; Di Fabio and Palazzeschi, 2016). While psychologists recognize the importance of systemic change to remedy these social and economic problems, they are also interested in identifying individual factors that foster well-being among future workers and can serve as assets that protect individuals from psychological harm and ultimately foster well-being and performance in the workplace (DeNeve and Cooper, 1998; Friedman and Kern, 2014; Gori et al., 2015).

Researchers (Antonovsky, 1987a; Seligman and Csikszentmihalyi, 2000) in the field of positive psychology recognize that optimal human functioning requires more than the absence of risk or pathology and have been seeking to identify factors that contribute to the development of hedonic and eudaimonic well-being (Ryan and Deci, 2001; Ryff and Singer, 2008; Waterman et al., 2010). Hedonic well-being entails the realization of happiness, pleasure attainment and pain avoidance, while eudaimonic well-being refers to the fulfillment or actualization of one’s full potential (Ryan and Deci, 2001). Hedonic well-being has been defined in research as subjective well-being (SWB) (Kahneman et al., 1999), operationalized as the prevalence of positive affect (PA) over negative affect (NA). In addition to these affective dimensions of SWB, life satisfaction is often considered a cognitive component of SWB (Pavot and Diener, 1993; Diener et al., 1999), although there is some debate about whether a precise definition of SWB should be restricted to affective indicators (Diener et al., 1999; Deci and Ryan, 2008). Lent (2004), however, suggests that life satisfaction entails both affective and cognitive dimensions, which has been supported by research (e.g., Arrindell et al., 1991; Lucas et al., 1996; Keyes et al., 2002) documenting moderate to strong relationships between positive–negative affect and life satisfaction. Research by Keyes et al. (2002) supports the conceptualization of PWB – psychological well-being and SWB as conceptually distinct constructs and the inclusion of life satisfaction as a dimension of SWB, despite some overlap across several indicators of SWB and PWB.

Eudaimonic well-being, referred to in research as both PWB (Ryff and Singer, 2008) and EWB – eudaimonic well-being (Waterman et al., 2010), emphasizes personal growth, mastery, life purpose and meaning (Ryff and Singer, 2008). Although a sense of SWB often accompanies PWB, pleasureful activities are considered insufficient to nurture and sustain PWB in the long-term (Ryan and Deci, 2001). PWB thus goes beyond the attainment of SWB (Ryff and Singer, 2008) and is derived from a sense of fulfillment, meaning, and self-realization. Research has found PWB to be broadly associated with indices of physical and mental health (Deci and Ryan, 2000) and is recognizably relevant to prevention, health promotion, and vocational psychology for its attention to selecting life goals and activities that are congruent with the true self and with deeply held values. To the extent that work is a source of identity, meaning, and personal connection (Blustein, 2006), work uncertainty and instability threaten both SWB and PWB.

Emotional intelligence (EI) has gained attention as a focus of research and intervention for its promise as a set of skills that can be taught to enhance coping resources and promote well-being (Schutte et al., 2007; Martins et al., 2010; Sánchez-Álvarez et al., 2015, 2016; Fernández-Berrocal, 2016). The connection between EI and a range of positive outcomes across the academic, social, psychological and career domains among adolescents has been well-documented (Di Fabio et al., 2014; Perera and DiGiacomo, 2015). Research has also found EI to be associated with a variety of individual and social resources, such as resilience, positive self-evaluation, and social support (Di Fabio and Kenny, 2012a; Di Fabio and Saklofske, 2014b; Perera and DiGiacomo, 2015). With regard to intervention, Di Fabio and Kenny (2011) found that a 10-h school-based intervention was effective in increasing EI and reducing career indecision among Italian high school students. Research with young adults in Belgium also demonstrated that EI can be increased with training, with EI gains sustained over a 6 months period (Nelis et al., 2009). Subsequent research applying the same EI intervention with Belgian university students revealed that intervention gains in EI led to positive changes in personality characteristics (increased agreeableness and extraversion and decreased neuroticism) assessed 6 months following intervention. Gains in psychological well-being, subjective health, quality of social relationships, and employability as reported by a prospective employer were also demonstrated at completion of the intervention in comparison with students receiving a different intervention or no intervention (Nelis et al., 2011).

A significant association between EI and well-being is supported by conceptual models that explain the possible causal mechanisms through which EI might influence well-being and by existing research that document some of those relationships. In a review of existing literature pertaining to EI, health, and well-being, Zeidner et al. (2012) suggest that EI influences SWB by fostering adaptive methods of coping with social challenges, social stress and interpersonal conflicts; promoting the development of supportive social networks; decreasing negative and increasing positive emotions; and enhancing emotional regulation. EI is also conceptually related to the PWB focus on personal growth and self-actualization (Zeidner et al., 2012). Skills in interpersonal (social) and intrapersonal (emotional awareness and internal self-regulation) EI should contribute to positive relationships with others and the capacity for mastery over one’s environment that allow for personal growth, a sense of meaning in life, and self-actualization (Zeidner and Olnick-Shemesh, 2010; Friedman and Kern, 2014). These conceptualizations of the pathways between EI and well-being are supported by studies documenting relationships between EI and social support (e.g., Di Fabio and Kenny, 2012a; Perera and DiGiacomo, 2015) and between EI and coping efficiency, stress reduction and emotional regulation (Mikolajczak et al., 2008, 2009).

Emotional intelligence has been conceptualized and assessed according to several different models and numerous measures, which has contributed to some controversy and complexity in the literature. Stough et al. (2009) identify the two primary approaches to defining and measuring EI as ability-based (Mayer and Salovey, 1997) and trait-based self-report (Bar-On, 1997; Petrides and Furnham, 2000, 2001). The ability-based model conceptualizes EI as a set of skills best assessed by performance-based problem-solving measures (Austin and Saklofske, 2014). The Mayer–Salovey–Caruso Intelligence Scale (MSCEIT; Mayer et al., 2002) is a frequently utilized performance measure of ability-based EI that assesses skills in the perception, understanding and management of emotions and the integration of emotion and cognition to enhance problem solving.

The trait model views EI as an emotion and personality-related disposition that is best assessed by self-report questionnaires. A number of trait-based self-report EI measures are available, with the Bar-On Emotional Quotient Inventory (EQ-i; Bar-On, 1997) and the Trait Emotional Intelligence Scale (TeiQue Petrides and Furnham, 2000, 2001) being among the most common. The Bar-On model is sometimes referred to as a “mixed-model” that includes self-reports of social-emotional personality dimensions and self-competencies (Bar-On, 1997), such as adaptability, stress-management, intrapersonal, and interpersonal awareness. The TEIQue focuses on self-perceptions of social-emotional personality factors, such as self-control, emotionality, and sociability. The TEIQue includes a dispositional measure of well-being entailing self-esteem, trait happiness, and trait optimism and the Bar-On includes an assessment of general mood that overlap with dispositional measures of personality and have some overlap with, but are not identical with state measures of affect or well-being (Mikolajczak et al., 2009). Moderate correlations have been found among various trait EI measures (Brackett and Mayer, 2003), suggesting that they overlap but are not identical (Freudenthaler et al., 2008).

Debate has focused on the extent to which EI is best understood and assessed as a set of emotion-focused abilities or as a blend of personality traits and social and emotional abilities (Zeidner et al., 2012). Ability-based and self-report trait measures of EI have been found to be generally uncorrelated, with overlap emerging only across subscales or dimensions of ability-based and trait-based measures that relate to similar affective content (Brackett and Mayer, 2003; Parker et al., 2011). For example with regard to overlap, persons who rate themselves positively on intrapersonal EI as assessed by trait-based measures also performed well in identifying and describing their own feelings and were knowledgeable about the influences of specific moods and emotions on reasoning and behavior as assessed through ability-based measure (Parker et al., 2011). The lack of overlap between trait and ability measures has been understood as a function of method variance related to different assessment methods (self-report vs. performance) and as a function of different individual difference domains (intelligence vs. personality) (Parker et al., 2011). Although both ability-based and trait EI constructs have some association with measures of personality and fluid intelligence, ability-based models assessed by performance measures have been found to overlap more with fluid intelligence, and trait models assessed by self-report measures overlap more with personality measures (Di Fabio and Palazzeschi, 2009). With regard to method variance, it is possible that the trait models of EI correlate with self-reports of personality because both measures assess positive self-perceptions (Zeidner et al., 2012). Although some scholars view the limited overlap between trait and ability measures as an indication of incompatibility of the two approaches (Mayer et al., 2008), other scholars argue that the two approaches focus on unique and complementary dimensions of EI (Austin et al., 2008; Mikolajczak et al., 2008; Parker et al., 2011).

To analyze the validity of EI as a construct distinct from personality and intelligence, programmatic research has assessed the contributions of both ability-based EI as assessed through the MSCEIT and trait models of EI assessed through the EQi and TeiQue to a variety of academic, social and career outcomes, controlling for the effects of fluid intelligence and personality. For example, both ability-based and trait measures explain academic grade point for high school and college students beyond the effects of fluid intelligence and personality, with ability-based measures explaining a greater share of the variance than self-report trait measures (Di Fabio and Palazzeschi, 2009). Perceived social support was also related, aside from the influences of fluid intelligence and personality, with both ability-based and trait EI, although trait EI was the more robust predictor for this outcome (Di Fabio and Kenny, 2012a). Studies of career decision-making also found both ability-based and trait EI to be significant predictors, but that trait EI was more robust than ability-based EI in explaining varied dimensions of decision-making (Di Fabio and Kenny, 2012b; Di Fabio and Saklofske, 2014a). Because both trait and ability-based EI explain academic, social, and career outcomes beyond the contributions of personality, evidence suggests that EI is distinct from the major dimensions of personality. Moreover, overall findings suggest that when dependent variables are self-reported, trait EI is the stronger predictor (Di Fabio and Kenny, 2012b; Di Fabio and Saklofske, 2014b), and that when the dependent variables are performance measures, ability-based EI explains more variance than self-report (Di Fabio and Palazzeschi, 2009). As mentioned above, the latter findings may be attributed to method variance as well as conceptual distinctions in EI as a personality construct and type of intelligence (Parker et al., 2011).

An emerging body of research has begun to examine relationships between EI and indices of hedonic well-being and eudaimonic well-being. The limited literature reveals some consistencies, but is also complicated by the use of varied measures of EI and well-being among different population samples. With regard to the relationships between self-reported trait EI and hedonic well-being, for example, research has consistently identified significant and positive relationships across studies of university students in Spain and community samples in Australia (Palmer et al., 2002; Extremera and Fernández-Berrocal, 2005; Gannon and Ranzijn, 2005; Gignac, 2006; Gallagher and Vella-Brodrick, 2008). Among high school students in Italy, Di Fabio and Saklofske (2014b) found that hedonic well-being was associated with trait measures of EI, but not with ability-based measures. Studies of trait EI and eudaimonic well-being are limited, but reveal positive findings. For example, an association between self-report measures of EI and eudaimonic well-being has been documented among professional employees in India (Raina and Bakhshi, 2013). Tennant et al. (2007) developed a new measure of EI, including items reflecting both eudemonic and hedonic domains, that was correlated with self-report measures of EI among university students in the United Kingdom. Perera and DiGiacomo (2015) found that trait EI was related to well-being, as measured by the Tennant et al. (2007) instrument. Evidence was found for a direct relationship and an indirect relationship mediated through perceived social support and engagement coping.

Findings for ability-based EI are more limited and reveal inconsistencies. Whereas Di Fabio and Saklofske (2014b) found no significant association between hedonic well- being and ability-based EI as assessed by the MSCEIT among Italian high schools students, Burrus et al. (2012) found a robust association between ability-based EI and hedonic and eudaimonic well-being for a US. sample of college and university students. Burrus et al. (2012) used a newly developed performance measure of emotional management, the Situational Judgment Test of Emotion Management (STEM; MacCann and Roberts, 2008), for assessing ability-based EI. The STEM presents participants with brief scenarios describing emotional situations that could potentially elicit negative emotions and asks participants to choose which of four options offered would be the best approach for managing the given situation. In a study of Spanish university students, Extremera et al. (2011) found that ability-based EI as assessed with the well-established MSCEIT measure was related with concurrent levels of both hedonic and eudaimonic well-being and with gains over a 12-week time period for both well-being constructs. Moreover, the gains in well-being explained by ability-based EI were greater for eudaimonic than for hedonic well-being. Inconsistencies in existing research are difficult to assess given the variations in measures of EI used across varied samples. As noted by Zeidner et al. (2012) in their research review, a systematic and fine grained approach to measurement of both criterion and predictor variables may be helpful in offering clarification for the field and providing a research base for preventive intervention.

The current study was designed to replicate and clarify understanding of the associations between EI and eudaimonic and hedonic well-being among young people preparing for work and further education, to inform the knowledge base for developing appropriate competencies for healthy personal and work lives (Di Fabio and Saklofske, 2014b; Di Fabio and Kenny, 2015). This study parallels prior research that examines the distinct contribution of EI by controlling for both fluid intelligence and personality (e.g., Di Fabio and Palazzeschi, 2009; Extremera et al., 2011; Di Fabio and Saklofske, 2014a). Controlling for personality, for example, is desirable as a method for accounting for the overlap between trait measures of EI and self-reports of personality (Zeidner et al., 2012). This study adds systematically and specifically to the limited existing research on hedonic and eudaimonic well-being by assessing for a single sample the relative contribution of ability-based EI and two models of trait EI, in explaining two types of well-being. The findings extend current research by using two well-established measures of self-report trait EI and a well-established performance measure of ability-based EI in explaining hedonic and eudaimonic well-being for students attending Italian high schools. Based upon theoretical conceptualization and the significant findings observed in prior research assessing self-reported trait and ability models of EI, we hypothesized that:

H1. Ability-based EI (Mayer et al., 2002) and trait EI based on both the Bar-On (1997) and the Petrides and Furnham (2000, 2001) and models will explain a significant percentage of incremental variance beyond the variance explained by fluid intelligence and personality traits in relation to hedonic well-being (PA, NA and life satisfaction).
H2. Ability-based EI (Mayer et al., 2002) and trait EI based on both the Bar-On (1997) and the Petrides and Furnham (2000, 2001) models will explain a significant percentage of incremental variance beyond the variance explained by fluid intelligence and personality traits in relation to eudaimonic well-being (meaningfulness).

## Materials and Methods

### Participants

One hundred and fifty seven high school students, 46 males (29.30%) and 111 females (70.70%), attending their last year of high school in Tuscany participated in the study. Participants were mostly White and from middle-class families and ranged from 17 to 21 years of age (*M* = 18.31; *SD* = 0.54). Participants were recruited in a school system located in the province of Florence with different addresses: classical (12.7%), linguistic (38.2%), scientific (33.1%), psycho-socio-pedagogic (16.9%).

### Measures

#### Advanced Progressive Matrices (APM)

To assess fluid intelligence, the APM test (Raven, 1962) in the Italian version (Di Fabio and Clarotti, 2007) was administered. The test has two series of items: Series I composed of 12 items and Series II composed of 36 items. Participants are asked to choose one correct response from eight possible options for each item. The Cronbach’s alpha as reported by Di Fabio and Clarotti (2007) was 0.91, and the alpha for the current study was 0.90.

#### Big Five Questionnaire (BFQ)

To assess personality traits, the (Caprara et al., 1993) was administered. The questionnaire has 132 items assessing five personality dimensions, with response options on a 5-point Likert scale format, from 1 = *Absolutely false* to 5 = *Absolutely true*. Reliability of the questionnaire scales was examined by using Cronbach’s alpha coefficient that ranged from a value of 0.73 for Agreeableness to 0.90 for Emotional Stability. The Cronbach’s alpha coefficients are: 0.81 for Extraversion, 0.73 for Agreeableness, 0.81 for Conscientiousness, 0.90 for Emotional Stability, and 0.75 for Openness.

#### Mayer–Salovey–Caruso Emotional Intelligence Test (MSCEIT)

To assess ability-based EI, the Mayer Salovey Caruso Emotional Intelligence Test (Mayer et al., 2002) in the Italian version (D’Amico and Curci, 2010) was administered. The measure has 141 items and provides a total score and four subscale scores: Perceiving Emotions (PE), Facilitating Thought (FT), Understanding Emotions (UE), and Managing Emotions (ME). The total score was used in the current study. Split half reliabilities for subscales reported by D’Amico and Curci (2010) are: 0.72 for ME, 0.75 for UE, 0.77 for FT, 0.90 for PE (D’Amico and Curci, 2010). The split half reliability for the total score for the current sample was 0.83.

#### Bar-On Emotional Intelligence Inventory (Bar-On EQ-i)

To assess trait EI, the Bar-On Emotional Intelligence Inventory (Bar-On EQ-i, Bar-On, 1997) in the Italian version (Franco and Tappatà, 2009) was administered. The questionnaire has 133 items with Likert scale response options ranging from 1 = *Not at all true of me* to 5 = *Absolutely true for me*. The measure yields a total Emotional Quotient (QE) and scores for five subscales: Intrapersonal, Interpersonal, Adaptability, Stress Management, and General Mood. Reliabilities were examined using Cronbach’s alpha coefficient for the Italian version of the EQ-I and they are the following: 0.95 for the total score, 0.91 for Intrapersonal, 0.84 for Interpersonal, 0.81 for Adaptability, 0.87 for Stress Management, 0.83 for General Mood (Franco and Tappatà, 2009).

#### Trait Emotional Intelligence Questionnaire (TeiQue)

The Italian version (Di Fabio, 2013) of the TeiQue (Petrides and Furnham, 2004) was also used assess trait EI. The questionnaire has 153 items with Likert scale response options ranging from 1 = *Completely disagree* to 7 = *Completely agree*. The measure provides a total score, and four subscale scores: Well-being, Self-Control, Emotionality, and Sociability. Cronbach’s alpha coefficient for the total score in the Italian version was 0.96 (Di Fabio et al., 2016). Cronbach’s alpha coefficients for the four subscales are also adequate and they are the following: 0.93 for Well-being, 0.81 for Self-Control, 0.92 for Emotionality, and 0.80 for Sociability (Di Fabio et al., 2016). The alpha for the total score for the current sample was 0.92.

#### Positive and Negative Affect Schedule (PANAS)

Positive Affect and Negative Affect were assessed as affective components of hedonic well-being using the Italian version (Terracciano et al., 2003) of the PANAS (Watson et al., 1988). The measure consists of 20 adjectives, 10 referring to PA (i.e., *enthusiastic, interested, determined*) and 10 referring to NA (i.e., *afraid, upset, distressed*). A participant specifies the intensity of affect experienced, using a Likert scale ranging from 1 = *Very slightly or not* at all to 5 = *Extremely*. The Cronbach’s alpha was 0.72 for PA and 0.83 for NA.

#### Satisfaction With Life Scale (SWLS)

Life satisfaction was also assessed as a component of hedonic well-being, consistent with the argument that life satisfaction entails an affective assessment of feelings about one’s life (Lent, 2004), using the Italian version (Di Fabio and Gori, 2015) of the Satisfaction With Life Scale (SWLS, Diener et al., 1985). The questionnaire consists of five items, which are rated using a 7-point Likert scale that ranges from 1 = *Strongly disagree* to 7 = *Strongly agree*. The Cronbach’s alpha coefficient reported by Di Fabio and Gori (2015) was 0.85, and for the current sample, the Cronbach’s alpha was 0.90.

#### Meaningful Life Measure (MLM)

Life meaningfulness was measured as a component of eudaimonic well-being, using the Italian translation (Di Fabio, 2014) of the MLM (Morgan and Farsides, 2009). The questionnaire consists of 23 items with a Likert response format ranging from 1 = *Strongly disagree* to 7 = *Strongly agree*. The scale assesses five dimensions of life meaningfulness: Exciting life, Accomplished life, Principled life, Purposeful life, Valued life. Cronbach’s alpha coefficients for the five dimensions are adequate for the Italian version: 0.87 for Exciting life to, 0.86 for Accomplished life, 0.85 for Principled life, 0.84 for Purposeful life, 0.87 for Valued life (Di Fabio, 2014). The Cronbach’s Alpha for the total score used in this study was 0.85.

### Procedure and Data Analysis

Questionnaires were administered, following Italian laws for privacy, by a trained research assistant to students in their high school classes. The administration of the questionnaires adhered to the requirements of privacy and informed consent in Italian law (Law Decree DL-196/2003) and the ethical standards for research of the Declaration of Helsinki revised in Fortaleza (World Medical Association [WMA], 2013), followed and approved by the Department of Education and Psychology of the University of Florence (Italy). Informed consent was recollected for each participant. Questionnaires were administered in counterbalanced order to control for ordering effects. Ten-minute breaks were provided after the APM and after the BFQ to limit fatigue.

In reference to preliminary data, the following analyses were performed: descriptive statistics, including means and standard deviations.

The Pearson’s correlation was then conducted in order to investigate the relation between the variables under investigation.

Eight separate hierarchical regression analyses were completed to examine predictors of hedonic and eudaimonic well-being, with PA, NA, and life satisfaction as separate indices of hedonic well-being, and with life meaningfulness as the index of eudaimonic well-being. For all analyses, fluid intelligence was entered at the first step, personality traits at the second step, and ability-based EI at the third step. For step four, trait EI assessed through TeiQue was entered in four of the analyses and trait EI assessed through EQ-i was entered for the other four analyses. In evaluating the accuracy of regression analyses standard errors and confidence intervals were considered. There were no missing data in the data set.

## Results

Means, standard deviations and correlations among APM, BFQ, MSCEIT, TeiQue, Bar-On EQ-I, PANAS PA, PANAS NA, SWLS, MLM are reported in **Table 1**.

**Table 1:** Means, standard deviations and correlations relative to APM, BFQ, MSCEIT, TeiQue, Bar-On EQ-i, PANAS PA, PANAS NA, SWLS, MLM.

	***M***	***DS***	**1**	**2**	**3**	**4**	**5**	**6**	**7**	**8**	**9**	**10**	**11**	**12**	**13**
	
(1) APM	34.94	6.57	–												
(2) Extraversion	76.02	10.63	0.03	–											
(3) Agreeableness	76.26	9.21	0.00	0.06	–										
(4) Conscientiousness	78.96	9.63	0.04	0.26**	0.03	–									
(5) Emotional Stability	61.69	13.81	0.08	0.04	0.26**	0.04	–								
(6) Openness	79.25	9.35	0.09	0.36***	0.30***	0.30***	0.09	–							
(7) MSCEIT	38.71	3.95	0.09	0.00	0.17*	0.14	0.04	0.23**	–						
(8) TeiQue	637.08	57.05	0.01	0.26**	0.20*	0.16*	0.23**	0.24**	0.09	–					
(9) Bar-On EQ-i	119.42	13.49	0.01	0.18*	0.40***	0.36***	0.27**	0.41***	0.29**	0.44**	–				
(10) PANAS PA	33.41	5.81	0.07	0.50***	0.01	0.37***	0.03	0.29***	0.01	0.30***	0.35***	–			
(11) PANAS NA	25.11	6.83	-0.11	-0.05	-0.17*	-0.03	-0.39***	-0.12	-0.04	-0.24**	-0.35***	-0.03	–		
(12) SWLS	20.71	6.07	0.04	0.29***	0.14	0.21**	0.30***	0.10	0.04	0.44***	0.32***	0.41***	-0.25*	–	
(13) MLM	101.09	17.00	0.12	0.36***	0.15	0.24**	0.00	0.23**	0.13	0.32***	0.35***	0.48***	-0.19*	0.42***	–

N = 157. *p < 0.05. **p < 0.01. ***p < 0.001. APM, Advanced Progressive Matrices; BFQ, Big Five Questionnaire; MSCEIT, Mayer–Salovey–Caruso Emotional Intelligence Test; TeiQue, Trait Emotional Intelligence Questionnaire; Bar-On EQ-I, Bar-On Emotional Quotient Inventory; PANAS PA, Positive and Negative Affect Schedule (Positive Affect); PANAS NA, Positive and Negative Affect Schedule (Negative Affect); SWLS, Satisfaction With Life Scale; MLM, Meaningful Life Measure.

In the analyses explaining hedonic well-being as assessed by PA, fluid intelligence was not significant in step one. In step two, personality traits explained a significant 32% of the variance; at the third step, ability-based EI was not significant; at the fourth step, trait EI assessed through the TeiQue explained an additional and significant 2% of the variance (**Table 2**). When trait EI assessed through the EQ-i was entered at the fourth step instead of trait EI assessed through the TeiQue, the fourth step explained an additional and significant 8% of the variance (**Table 4**).

**Table 2:** Hierarchical regression.

	PANAS PA								PANAS NA								SWLS							
	Unstd. est.		Std. est.			Confidence intervals			Unstd. est.		Std. est.			Confidence intervals			Unstd. est.		Std. est.			Confidence intervals		
	β	*SE*	β	*t*	*p*	inf	sup	Hierarchical regression	β	*SE*	β	*t*	*p*	inf	sup	Hierarchical regression	β	*SE*	β	*t*	*p*	inf	sup	Hierarchical regression
APM	-0.058	0.071	-0.066	-0.825	0.411	-0.198	0.082		-0.112	0.083	-0.108	-1.352	0.178	-0.276	0.052		0.038	0.074	0.041	0.514	0.608	-0.108	0.185	
*R2 step 1*								0.00								0.01								0.00
BFQ–E	0.227	0.041	0.415	5.577	0.000	0.146	0.307		-0.043	0.053	-0.067	-0.816	0.416	-0.147	0.061		0.177	0.046	0.310	3.868	0.000	0.086	0.267	
BFQ–A	0.010	0.047	0.016	0.213	0.832	-0.082	0.102		-0.050	0.060	-0.068	-0.833	0.406	-0.170	0.069		0.075	0.052	0.114	1.438	0.153	-0.028	0.179	
BFQ–C	0.144	0.043	0.238	3.316	0.001	0.058	0.229		0.056	0.056	0.079	0.999	0.319	-0.055	0.167		0.099	0.049	0.157	2.032	0.044	0.003	0.195	
BFQ–S	-0.012	0.029	-0.029	-0.419	0.676	-0.070	0.046		-0.181	0.038	-0.366	-4.744	0.000	-0.256	-0.106		0.126	0.033	0.287	3.820	0.000	0.061	0.192	
BFQ–M	0.045	0.049	0.072	0.907	0.366	-0.053	0.142		-0.046	0.064	-0.063	-0.718	0.474	-0.172	0.080		-0.081	0.055	-0.125	-1.462	0.146	-0.190	0.028	
*ΔR2 step 2*								0.32***								0.16***								0.21***
MSCEIT (EI ability-based)	-0.053	0.104	-0.036	-0.516	0.607	-0.258	0.151		-0.011	0.135	-0.006	-0.078	0.938	-0.277	0.256		0.028	0.117	0.018	0.242	0.809	-0.203	0.259	
*ΔR2 step 3*								0.00								0.00								0.00
‘TEIQUE tot (trait EI)	0.017	0.007	0.167	2.305	0.023	0.002	0.032		-0.017	0.010	0.010	-1.774	0.078	-0.036	0.002		0.035	0.008	0.332	4.438	0.000	0.020	0.051	
*ΔR2 step 4*								0.02*								0.02*								0.09***
*R2 total*								0.34***								0.19***								0.30***

N = 157. *p < 0.05. ***p < 0.001. The contributions of fluid intelligence (First step), personality traits (Second step), ability-based emotional intelligence Total (Third step) and trait emotional intelligence assessed through TeiQue Total (Fourth step) to positive affect (PANAS PA), negative affect (PANAS NA), life satisfaction (SWLS). APM, Advanced Progressive Matrices; BFQ, Big Five Questionnaire; E, Extraversion; A, Agreableness; C, Consientiousness; S, Emotional Stability; M, Openness; MSCEIT, Mayer–Salovey–Caruso Emotional Intelligence Test; TeiQue, Trait Emotional Intelligence Questionnaire; PANAS PA, Positive and Negative Affect Schedule (Positive Affect); PANAS NA, Positive and Negative Affect Schedule (Negative Affect); SWLS, Satisfaction With Life Scale.

In the analyses explaining hedonic well-being as assessed by NA, fluid intelligence was not significant in step one. At the second step, personality traits explained a significant 16% of the variance; at the third step, ability-based EI was not significant; at step four, trait EI assessed through the TeiQue explained an additional 2% of the variance (**Table 2**). When trait EI assessed through the EQ-i was entered at the fourth step instead of trait EI assessed through the TeiQue, the EQ-i explained an additional significant 8% of the variance (**Table 4**).

In the analyses assessing hedonic well-being as assessed by life satisfaction, fluid intelligence was not significant at the first step. At the second step, personality traits explained a significant 21% of the variance; at the third step, ability-based EI was not significant; at the fourth step, trait EI assessed through the TeiQue explained an additional and significant 9% of the variance (**Table 2**). When trait EI assessed through EQ-i was entered at the fourth step instead of trait EI assessed through TeiQue, the EQ-i explained a significant 7% of the variance (**Table 4**).

For the analyses assessing life meaningfulness as a component of eudaimonic well-being, fluid intelligence was not significant at the step one. At the second step, personality traits explained a significant 18% of the variance; at the third step, ability-based EI was not significant; at the fourth step, trait EI assessed through the TeiQue explained an additional significant 4% of the variance (**Table 3**). When trait EI assessed through the EQ-i was entered at the fourth step instead of trait EI assessed through the TeiQue, trait EI assessed through the EQ-i explained an additional and significant 10% of variance over step three (**Table 5**).

**Table 3:** Hierarchical regression.

	MLM							
	Unstd. est.		Std. est.			Confidence intervals		Hierarchical regression
	β	*SE*	β	*t*	*p*	inf	sup	
APM	0.325	0.206	0.125	1.575	0.117	-0.083	0.732	
*R2 step 1*								0.00
BFQ–E	0.509	0.130	0.318	3.923	0.000	0.253	0.765	
BFQ–A	0.312	0.148	0.169	2.104	0.037	0.019	0.606	
BFQ–C	0.267	0.138	0.151	1.930	0.055	-0.006	0.540	
BFQ–S	-0.066	0.094	-0.054	-0.705	0.482	-0.251	0.119	
BFQ–M	0.031	0.157	0.017	0.198	0.843	-0.279	0.341	
*ΔR2 step 2*								0.18***
EI ability-based	0.320	0.330	0.074	0.968	0.334	-0.333	0.972	
*ΔR2 step 3*								0.01
TEIQUE tot	0.067	0.023	0.225	2.875	0.005	0.021	0.113	
*ΔR2 step 4*								0.04**
*R2 total*								0.24***

N = 157. **p < 0.01. ***p < 0.001. The contributions of fluid intelligence (First step), personality traits (Second step), ability-based emotional intelligence Total (Third step) and trait emotional intelligence assessed through TeiQue Total (Fourth step) to life meaningfulness (MLM). APM, Advanced Progressive Matrices; BFQ, Big Five Questionnaire; E, Extraversion; A, Agreableness; C, Consientiousness; S, Emotional Stability; M, Openness; MSCEIT, Mayer–Salovey–Caruso Emotional Intelligence Test; TeiQue, Trait Emotional Intelligence Questionnaire; MLM, Meaningful Life Measure.

**Table 4:** Hierarchical regression.

	PANAS PA								PANAS NA								SWLS							
	Unstd. est.		Std. est.			Confidence intervals			Unstd. est.		Std. est.			Confidence intervals			Unstd. est.		Std. est.			Confidence intervals		
	β	*SE*	β	*t*	*p*	inf	sup	Hierarchical regression	β	*SE*	β	*t*	*p*	inf	sup	Hierarchical regression	β	*SE*	β	t	*p*	inf	sup	Hierarchical regression
APM	-0.058	0.071	-0.066	-0.825	0.411	-0.198	0.082		-0.112	0.083	-0.108	-1.352	0.178	-0.276	0.052		0.038	0.074	0.041	0.514	0.608	-0.108	0.185	
*R2 step 1*								0.00								0.01								0.00
BFQ–E	0.227	0.041	0.415	5.577	0.000	0.146	0.307		-0.043	0.053	-0.067	-0.816	0.416	-0.147	0.061		0.177	0.046	0.310	3.868	0.000	0.086	0.267	
BFQ–A	0.010	0.047	0.016	0.213	0.832	-0.082	0.102		-0.050	0.060	-0.068	-0.833	0.406	-0.170	0.069		0.075	0.052	0.114	1.438	0.153	-0.028	0.179	
BFQ–C	0.144	0.043	0.238	3.316	0.001	0.058	0.229		0.056	0.056	0.079	0.999	0.319	-0.055	0.167		0.099	0.049	0.157	2.032	0.044	0.003	0.195	
BFQ–S	-0.012	0.029	-0.029	-0.419	0.676	-0.070	0.046		-0.181	0.038	-0.366	-4.744	0.000	-0.256	-0.106		0.126	0.033	0.287	3.820	0.000	0.061	0.192	
BFQ–M	0.045	0.049	0.072	0.907	0.366	-0.053	0.142		-0.046	0.064	-0.063	-0.718	0.474	-0.172	0.080		-0.081	0.055	-0.125	-1.462	0.146	-0.190	0.028	
*ΔR2 step 2*								0.32***								0.16***								0.21***
EI ability-based	-0.053	0.104	-0.036	-0.516	0.607	-0.258	0.151		-0.011	0.135	-0.006	-0.078	0.938	-0.277	0.256		0.028	0.117	0.018	0.242	0.809	-0.203	0.259	
*ΔR2 step 3*								0.00								0.00								0.00
Eq-I Tot	0.117	0.036	0.272	3.293	0.001	0.047	0.187		-0.177	0.046	-0.350	-3.883	0.000	-0.267	-0.087		0.101	0.041	0.224	2.479	0.014	0.020	0.181	
*ΔR2 step 4*								0.08***								0.08***								0.07***
*R2 total*								0.40***								0.25***								0.29***

N = 157. ***p < 0.001. The contributions of fluid intelligence (First step), personality traits (Second step), ability-based emotional intelligence Total (Third step) and trait emotional intelligence assessed through EQ-i Total (Fourth step) to positive affect (PANAS PA), negative affect (PANAS NA), life satisfaction (SWLS). APM, Advanced Progressive Matrices; BFQ, Big Five Questionnaire; E, Extraversion; A, Agreableness; C, Consientiousness; S, Emotional Stability; M, Openness; MSCEIT, Mayer–Salovey–Caruso Emotional Intelligence Test; Bar-On EQ-I, Bar-On Emotional Quotient Inventory; PANAS PA, Positive and Negative Affect Schedule (Positive Affect); PANAS NA, Positive and Negative Affect Schedule (Negative Affect); SWLS, Satisfaction With Life Scale.

**Table 5:** Hierarchical regression.

	MLM							
	Unstd. est.		Std. est.			Confidence intervals		Hierarchical regression
	β	Standard error	β	*t*	*p*	inf	sup	
APM	0.325	0.206	0.125	1.575	0.117	-0.083	0.732	
*R2 step 1*								0.02
BFQ – E	0.509	0.130	0.318	3.923	0.000	0.253	0.765	
BFQ – A	0.312	0.148	0.169	2.104	0.037	0.019	0.606	
BFQ – C	0.267	0.138	0.151	1.930	0.055	-0.006	0.540	
BFQ – S	-0.066	0.094	-0.054	-0.705	0.482	-0.251	0.119	
BFQ – M	0.031	0.157	0.017	0.198	0.843	-0.279	0.341	
*ΔR2 step 2*								0.18***
EI ability-based	0.320	0.330	0.074	0.968	0.334	-0.333	0.972	
*ΔR2 step 3*								0.01
Eq-I tot	0.355	0.114	0.282	3.125	0.002	0.131	0.580	
*ΔR2 step 4*								0.10***
*R2 total*								0.31***

N = 157. ***p < 0.001. The contributions of fluid intelligence (First step), personality traits (Second step), ability-based emotional intelligence Total (Third step) and trait emotional intelligence assessed through EQ-i Total (Fourth step) to life meaningfulness (MLM). APM, Advanced Progressive Matrices; BFQ, Big Five Questionnaire; E, Extraversion; A, Agreableness; C, Consientiousness; S, Emotional Stability; M, Openness; MSCEIT, Mayer–Salovey–Caruso Emotional Intelligence Test; Bar-On EQ-I, Bar-On Emotional Quotient Inventory; MLM, Meaningful Life Measure.

## Discussion

The current study was designed to examine the contribution of EI, including ability-based EI and two measures of self-report trait EI, to indices of hedonic and eudaimonic well-being in young people, with concern from a prevention perspective for their well-being as future workers. Analyses were completed to assess the incremental variance, beyond the effects of fluid intelligence and personality traits, of ability-based EI and two different self-report EI models.

The overall findings partially support the two main hypotheses that focused on the contributions of EI, in explaining hedonic and eudaimonic well-being beyond the variance explained by fluid intelligence and personality traits. More specifically, trait EI, as assessed through the TeiQue (Petrides and Furnham, 2000, 2001) and the EQ-i (Bar-On, 1997), explained significant variance across the three indices of hedonic well-being and the MLM of eudaimonic well-being. These findings with Italian high school students thus confirm prior research documenting relationships between self-report measures of EI and hedonic well-being with Italian college students (Di Fabio and Saklofske, 2014b), with Spanish university students (Extremera and Fernández-Berrocal, 2005), and with community samples in Australia (Palmer et al., 2002; Gannon and Ranzijn, 2005; Gignac, 2006; Gallagher and Vella-Brodrick, 2008). The current results are also consistent with prior research that has found self-report measures of EI to be associated with eudaimonic well-being (Tennant et al., 2007; Raina and Bakhshi, 2013), indicating that self-ratings of one’s EI are related to the self-ratings of one’s life as meaningful and pleasant.

Our hypothesis that ability-based EI would be associated with both hedonic and eudaimonic well-being was not supported which conflicts with some prior findings. The discrepancy between the current and other findings might be understood by variations in measures used. Burrus et al. (2012), for example, used a new and unique measure of EI with US students. In their study of Spanish university students, Extremera et al. (2011) employed the well-established MSCEIT, as used in the current study for measuring ability-based EI, but employed a measure of well-being that was different than the current study. Our findings are consistent with previous research among Italian high school students, which found no relation between ability-based EI as assessed by the MSCEIT and hedonic well-being (Di Fabio and Saklofske, 2014b). Further research will be needed to discern whether the difference in our findings and the Extremera et al. (2011) findings are related to national, developmental, or measurement differences.

Our findings do confirm prevailing evidence that self-report measures of EI are more robust than ability-based EI in explaining well-being and other psychological constructs assessed through self-report (Zeidner et al., 2012). While this may partly be understood as an issue of method variance, we have also found that when EI is conceptualized affectively, rather than cognitively, EI is more strongly related to other self-perceptions, including well-being (Di Fabio and Kenny, 2012b; Di Fabio et al., 2014). Our finding that trait EI explains well-being beyond the variance explained by personality, however, suggests that trait EI cannot be understood simply as a broad based personality factor. While trait EI, as assessed by both the EQ-i and TeiQue, is clearly associated with trait dimensions of personality, such as extraversion, agreeableness, emotional stability, and openness assessed in this study, our findings and a preponderance of prior evidence (Di Fabio et al., 2014) suggest that trait EI goes beyond those factors in explaining a variety of important psychological outcomes. The current study expands that body of literature by affirming the contributions of two measures of trait EI beyond personality factors in explaining both hedonic and eudaimonic well-being and suggests that the two trait measures of EI contribute somewhat differently to well-being.

While the current study confirms prior research on the contributions of trait EI to other self-report psychological constructs, our findings also affirm complexities and variations based upon the self-report measures employed. The current results are most similar to research that has employed similar measures of trait EI (e.g., Di Fabio and Saklofske, 2014b), and illustrate that the extent to which trait EI explains hedonic well-being can vary depending on the conceptualization and choice of measure. In this study, the variance accounted for by the EQ-i was more robust than for the TeiQue in explaining dimensions of both hedonic and eudaimonic well-being. In prior research (Di Fabio and Saklofske, 2014a,b), the TeiQue was more robust, explaining twice as much variance as the EQ-i in relation to three career dimensions, resilience, and positive core self-evaluation. While the explanation for these differences in not clear, our findings highlight how results can vary across studies related to measures and selected outcome variables and that different measures and models of trait EI, while overlapping, are not identical. Although further research is necessary to replicate and expand the current findings, it is possible that the EI components incorporated in the EQ-i, such as adaptability, stress management, and general mood, are more aligned with well-being, and whereas self-control and sociability as assessed by the TeiQue are more aligned with career decision-making. Further research might examine these nuances.

Given the interest among proponents of positive psychology in the promotion of meaningfulness and optimal functioning in the work environment (Bakker and Schaufeli, 2008; Heuvel et al., 2010), the promise of EI as a contributor to eudaimonic well-being is noteworthy. Heuvel et al. (2010) emphasize the importance of identifying personal competencies/resources, as well as organizational factors, that enable employees to be happy, engaged and productive in the new and ever-changing workplace. Our findings suggest that the promotion of trait EI may function not only as a method for enhancing hedonic well-being, including PA, life satisfaction, and reduced NA, but may also serve to enhance a sense of a meaningful life. Given the threats posed to well-being by social and economic change and work uncertainty, factors associated with life meaning are worthy of continued research attention and consideration for intervention for fostering healthy individuals and organizations (Zeidner et al., 2012; Friedman and Kern, 2014; Snyder et al., 2014).

Despite the contribution of the current study in documenting the contributions of trait EI, based on both the Bar-On (1997) and the Petrides and Furnham (2000, 2001) models, for both hedonic and eudaimonic well-being, the limitations of the study should be considered. Our results are correlational and causality cannot be concluded. Further research is needed to determine how trait EI can best be enhanced and whether those gains can lead to gains in both hedonic and eudaimonic well-being. Longitudinal research is also needed to systematically test the causal mechanisms that promote well-being as a function of EI intervention. We have noted differences in prior research based upon sample characteristics, such that the findings of this study based on high school students in the Tuscany Region of Italy cannot be generalized to other samples. The current findings deserve replication with participants more representative of all Italians across geography and age and could be also replicated in other national contexts. We studied only one aspect of eudaimonic well-being, such that the relationship of EI to other components of eudaimonic well-being, such as existential fulfillment (Längle et al., 2003), sense of coherence (Antonovsky, 1987b), and authenticity (Wood et al., 2008), should also be studied. This study controlled for the association of EI and personality measures among the predictor variables, but other potential confounds between EI and the criterion measures (e.g., well-being and mood as a component of trait EI) need to be considered and controlled. The use of objective measures of well-being will help in this regard. While well-being is a subjective construct and thus reasonably assessed through self-report, further research should include more objective indices of well-being, including physical health, educational or work productivity, and creative achievements.

Despite the limitations of the current study, the findings add to and extend the literature highlighting the positive associations and potential benefit of diverse models of trait EI, suggesting promise of the construct for continued research in positive psychology (Seligman and Csikszentmihalyi, 2000) and positive organizational behavior (Bakker and Schaufeli, 2008). While continued research is needed to support the evidence base for EI intervention (Zeidner et al., 2012), trait EI could be a potential focus for enhancing the subjective and psychological well-being of young and future workers and for maintaining and fostering health and performance in a challenging work environment (Di Fabio, 2015; Di Fabio and Palazzeschi, 2015; Arnoux-Nicolas et al., 2016).

## Author Contributions

AD and MK conceptualized the study, chose the theoretical framework and chose the measures. AD collected the data and with MK wrote the methods and results. Then the authors wrote the paper together and read and revised the manuscript several times.

## Conflict of Interest Statement

The authors declare that the research was conducted in the absence of any commercial or financial relationships that could be construed as a potential conflict of interest.
